# Accidental Wounds of the Female Bladder

**Published:** 1899-11

**Authors:** Frederick Holme Wiggin

**Affiliations:** New York City


### ACCIDENTAL WOUNDS OF THE FEMALE BLADDER.

BY FREDERICK HOLME WIGGIN, M. D., NEW YORK CITY.

Presented to the Section on Obstetrics and Diseases of Women, at the Fiftieth Annual Meeting of the American Medical Association, held at Columbus, Ohio, June 6th-9th, 1899.

(Abstract from the *Journal of the American Medical Association*, of Sept. 9th, 1899.)

Accidental opening of the bladder has, for many years, been considered one of the most serious accidents that could occur in the course of the complicated work which gynecic surgeons are often called on to perform. The following case is offered in illustration of this type of injury:

M. H., unmarried, æt. 41, was admitted to the City Hospital, Blackwell's Island, N. Y., Sept. 30th, 1898, suffering from a large myoma, which sprung from the anterior uterine wall and extended above the umbilicus. On Oct. 3d, the abdomen was opened, and the tumor, which weighed seventeen pounds was drawn through an incision six inches in length, freed from its attachments and removed, together with the body of the uterus amputated near the internal os. As hemorrhage was profuse it became necessary to remove the mass very rapidly, to accomplish which the anterior attachment of the tumor was clamped and cut, when it was discovered, from the escape of urine, that the bladder had been opened near the fundus.

The general cavity had previously been shut off with gauze pads and thoroughly irrigated, followed by the use of Hydrozone in half strength, and this, in turn, by a saline solution. The gauze pads were now changed, and the opening in the bladder, four inches in length, was closed by means of two layers of chromicized catgut sutures. The wound was then disinfected, and there being a large peritoneal flap, it was attached to the bladder and made to cover the line of sutures, thus making the bladder-wound extra peritoneal. After further washing out of the abdominal cavity with Hydrozone and the saline solution the external wound was closed, without drainage, and the usual dressings applied. The patient being feeble it was not thought advisable to make a vesico-vaginal fistula to drain the bladder, but, instead, a self-retaining catheter was introduced. At the end of ten days, however, tumefaction occurred over the lower angle of the abdominal wound, and, on opening it, urine began to escape. A vesico-vaginal fistula was now made in order to afford adequate drainage. The sinus in the abdominal wall was curetted and, after being thoroughly disinfected with Hydrozone, its walls were sutured. Soon afterwards, the sinus having closed, the sutures which kept open the vesico-vaginal fistula were removed, and the latter closed quickly without any further operative interference.

Percival (in *British Medical Journal*, 1897, Vol. 1, p. 1282)

reports a case of ruptured bladder on which he had operated. It was closed by means of a double wall of Lembert silk sutures. The wound in the abdominal wall was closed, after the peritoneal cavity had been flushed out with boric acid solution and a large quantity of clots and urinous fluids had been removed. For a few days the patient did well, and then died from peritonitis. But the necropsy proved that the bladder-wound had completely healed. It is the writer's opinion that had saline solution and Hydrozone been used, instead of boric acid, and the abdominal wound been closed leaving saline solution in the peritoneal cavity the patient would probably have recovered.

